# Learning Online MEMS Calibration with Time-Varying and Memory-Efficient Gaussian Neural Topologies

**DOI:** 10.3390/s25123679

**Published:** 2025-06-12

**Authors:** Danilo Pietro Pau, Simone Tognocchi, Marco Marcon

**Affiliations:** 1 System Research and Applications, STMicroelectronics, Via C. Olivetti 2, 20864 Agrate Brianza, Italy; simone.tognocchi@st.com; 2 Department of Electronics, Information and Bioengineering, Politecnico di Milano, 20133 Milan, Italy; marco.marcon@polimi.it

**Keywords:** on-device learning, radial basis functions, inertial sensor calibration, intelligent sensor processing unit, in-sensor tiny machine learning

## Abstract

This work devised an on-device learning approach to self-calibrate Micro-Electro-Mechanical Systems-based Inertial Measurement Units (MEMS-IMUs), integrating a digital signal processor (DSP), an accelerometer, and a gyroscope in the same package. The accelerometer and gyroscope stream their data in real time to the DSP, which runs artificial intelligence (AI) workloads. The real-time sensor data are subject to errors, such as time-varying bias and thermal stress. To compensate for these drifts, the traditional calibration method based on a linear model is applicable, and unfortunately, it does not work with nonlinear errors. The algorithm devised by this study to reduce such errors adopts Radial Basis Function Neural Networks (RBF-NNs). This method does not rely on the classical adoption of the backpropagation algorithm. Due to its low complexity, it is deployable using kibyte memory and in software runs on the DSP, thus performing interleaved in-sensor learning and inference by itself. This avoids using any off-package computing processor. The learning process is performed periodically to achieve consistent sensor recalibration over time. The devised solution was implemented in both 32-bit floating-point data representation and 16-bit quantized integer version. Both of these were deployed into the Intelligent Sensor Processing Unit (ISPU), integrated into the LSM6DSO16IS Inertial Measurement Unit (IMU), which is a programmable 5–10 MHz DSP on which the programmer can compile and execute AI models. It integrates 32 KiB of program RAM and 8 KiB of data RAM. No permanent memory is integrated into the package. The two (fp32 and int16) RBF-NN models occupied less than 21 KiB out of the 40 available, working in real-time and independently in the sensor package. The models, respectively, compensated between 46% and 95% of the accelerometer measurement error and between 32% and 88% of the gyroscope measurement error. Finally, it has also been used for attitude estimation of a micro aerial vehicle (MAV), achieving an error of only 2.84°.

## 1. Introduction

Micro-Electro-Mechanical Systems-based Inertial Measurement Units are widely used in a broad range of applications due to their compact size, low power consumption, and cost-effectiveness [1]. These advantages have contributed to their widespread adoption across various fields, such as consumer electronics [2], where they are integrated components in smartphones, tablets, and wearable devices. Their small form factor and low cost make them ideal for enhancing user–machine interaction through motion detection and orientation measurements. In the healthcare sector [3], MEMS IMUs play a crucial role in motion tracking, rehabilitation, and fall detection systems. Their ability to monitor human motion in a non-invasive manner enables continuous assessment and diagnostics, particularly in elderly care and physiotherapy. In aerospace applications [4], IMUs contribute to the navigation and control systems of aircraft, spacecraft, and drones, where reliable attitude and motion information are critical for stability and mission success. The automotive industry and navigation systems also benefit significantly from IMUs [5,6], employing them in advanced driver-assistance systems (ADAS), vehicle dynamics monitoring, and autonomous navigation where GPS signals may be unavailable or degraded by noise.

These devices integrate three-axial accelerometers and gyroscopes based on MEMS technology. This integration allows for the measurement of linear acceleration and angular velocity across three orthogonal axes, enabling full 3D motion tracking. MEMS technology enables miniaturization and efficient energy use while maintaining a balance between cost and performance. This combination of characteristics makes IMUs suitable not only for high-end industrial applications but also for large-scale deployment in consumer-grade devices.

However, despite these benefits, MEMS IMUs are affected by some limitations. The accuracy and reliability of their measurements can degrade over time due to several error sources [7,8]. These include scale factor errors, time-varying bias, cross-axis sensitivity, temperature-dependent drift, misalignment errors, and random measurement noise. Unlike high-end fiber optic or ring laser gyroscopes, MEMS-based sensors are more susceptible to environmental factors, which introduces challenges for long-term precision and stability.

Many of these errors are introduced during the manufacturing and assembly processes. Fabrication defects and imperfections in the mechanical structures of the MEMS sensors can lead to deviations from ideal behavior [9]. Mounting misalignment of the accelerometer triads with respect to the sensor package can cause cross-axis sensitivities and orientation errors. Additionally, thermal stress, such as that experienced during deployment in environments with fluctuating temperatures, can induce structural changes in the MEMS elements, leading to drift and non-linearity in sensor outputs [10].

Given these factors, the accuracy of MEMS IMU measurements can degrade if not properly corrected. Over time, this can lead to significant errors in motion estimation and navigation computations, especially in applications requiring long-term stability and precision. To mitigate these effects and ensure reliable performance, periodic calibration of IMUs is essential. This calibration should be performed by the sensor itself through self-calibration algorithms, which can operate without requiring external references, in order to reduce the need for manual recalibration procedures and enhance the adaptability of IMUs in dynamic and uncontrolled environments.

This paper is structured as follows. Section 2 reviews the existing literature on MEMS-IMU calibration techniques and on-device learning methods. Section 3 defines the problem addressed in this work and outlines the requirements for an effective solution. The main contributions of this study are summarized in Section 4. Section 5 describes the proposed approach in detail, while Section 6 explains its implementation on the ISPU. The datasets used for evaluation are introduced in Section 7, followed by the definition of the performance metrics in Section 8. The experimental setup and results obtained on both datasets are presented in Section 9. Section 10 discusses the findings and their implications, and finally, Section 11 concludes the paper with a summary of the work and suggestions for future research directions.

## 2. Related Works

The related work section provides an overview of the existing literature on MEMS calibration approaches, starting from the traditional ones, then presenting the ones using Deep Learning (DL) and Artificial Neural Networks (ANN), and, finally, introducing the literature about on-device learning using tiny Machine Learning (tinyML) and forward-only models.

### 2.1. MEMS Calibration Approaches

#### 2.1.1. Traditional Approaches

Previous research on the calibration of tri-axial MEMS IMUs has been based on a unified error model and an ellipsoid fitting algorithm utilizing direct least squares (DLS) to estimate the error model parameters for calibration [11]. However, this approach is limited to scenarios without interference errors and under static conditions.

In [12] is proposed an automated self-calibration method for land navigation systems estimating the spatial misalignment between an IMU and vehicle frame using data from an IMU, uncalibrated odometer, and GPS. It performs an observability analysis to identify conditions and maneuvers that make calibration parameters reliably estimable. This removes the need for manual calibration and leverages existing onboard sensors. The main limitations are its dependence on executing specific vehicle maneuvers to ensure observability and the assumption of reliable odometer and GPS data.

Other studies have attempted to use the Kalman Filter (KF) to develop a real-time, low-cost MEMS calibration method without external equipment or temperature data dependency [13]. While effective in linear contexts with reduced computational load, the KF is inadequate for nonlinear compensation. The Extended Kalman Filter (EKF) improves upon this by linearizing nonlinear models around the current estimate, enabling real-time estimation in mildly nonlinear systems [14]. However, EKF performance degrades with strong nonlinearities or poor initial estimates, and its reliance on Jacobian computations adds complexity and potential instability.

Ghanipoor et al. [15] proposed a method employing the Transformed Unscented Kalman Filter (TUKF) to estimate error parameters for gyroscopes and accelerometers. The TUKF approach was able to provide a relatively robust solution for the estimation of error parameters for both gyroscopes and accelerometers but required a tri-axis turntable to generate input signals, which presents a significant limitation to the method.

#### 2.1.2. Machine Learning-Based Approaches

Recently, several methods using deep learning (DL) and Artificial Neural Networks (ANNs) for thermal calibration of the bias have been proposed [16,17], but without solving the aging problem.

OriNet [18] attempted to estimate the complete three-dimensional orientation of a flying robot using a single IMU and multiple long short-term memory (LSTM) layers. Although this approach yielded favorable results on the EuRoC MAV Dataset, it, like all methods employing LSTM or gated recurrent units (GRU), requires high computational complexity and is thus less desirable for resource-constrained devices and real-time applications.

Other studies have attempted to bypass the use of recurrent neural networks (RNN), such as [19], by employing an approach based on neural networks utilizing dilated convolutions. Also, this study achieved remarkable accuracy on the EuRoC MAV Dataset with less computational effort than OriNet, though it still involved approximately 77,000 trainable parameters. The primary limitation of this solution is its offline nature, as it cannot be implemented on resource-constrained sensors for online calibration. Additionally, this work required an appropriate dataset for training.

Another study that adopted dilated convolutions for spatio-temporal feature extraction of IMU measurements instead of recurrent neural networks was CalibNet [20]. This work implemented a more lightweight deep convolutional neural network (CNN) that outperformed [19], yet it remains unsuitable for on-device learning. Ref. [21] developed a Temporal Convolutional Network (TCN) that achieved more accurate attitude estimation compared with DIG and OriNet. However, the TCN used in this study contained over 175,000 trainable parameters, necessitating substantial computational resources, particularly during training.

Recently, TinyGC-Net [22] effectively implemented two networks with only 27 and 195 trainable parameters, comprising two linear networks linked by a Parametric Rectified Linear Unit (PReLU). These networks performed better on the EuRoC MAV Dataset than [18], although not as well as [19]. Nonetheless, they utilized significantly fewer parameters, thereby reducing computational complexity considerably. Despite this, the network training was still conducted offline using a Graphics Processing Unit (GPU).

Another study on the calibration of MEMS IMU accelerometers is [23], which introduced four distinct tiny Convolutional Neural Networks (CNNs). Two of these networks operate with 32-bit floating-point precision, while the other two employ a 16-bit quantization–aware training approach. Each network contains fewer than 1000 parameters and exhibits strong performance on the same field dataset used in this work. The best model among the four has been used as a benchmark for comparing some of the results of the model devised in this study.

### 2.2. On-Device Learning

All the AI models presented, including tinyML models, offer promising solutions for sensor calibration with high accuracy. However, they require on-premises or cloud training using costly and power-hungry GPUs, and this type of learning cannot be deployed on low-power sensor chips with limited resources. Moreover, AI models intended for accurate sensor calibration over time cannot be trained offline due to the drift problem [24], which can degrade performance when data distribution or operating conditions change. Thus, it is essential to find a sensor calibration solution capable of adapting its learning in real time to maintain consistent accuracy, even with changing environmental conditions. Various tinyML approaches have been proposed [25] to primarily deploy models for inference on devices with limited resources. These models are optimized offline and then implemented on a microcontroller unit (MCU) to perform inference on sensor-acquired data. However, these models encounter challenges due to their static nature and lack of adaptability to environmental changes. An explored idea is the use of reformable online learning [26], a TinyML solution that can enhance itself through local and remote/over-the-air updates. Nonetheless, while many of these systems operate with relatively relaxed device constraints, algorithms designed for more resource-constrained environments often rely on fine-tuning the initial model, limiting the potential for fully autonomous online learning [27].

A key concept for addressing the stringent requirements of on-device sensor calibration within the sensor package is to utilize AI models capable of consistent online self-learning over time to tackle the drift problem and adapt the model in real-time to changes in data. To achieve this, it is essential to avoid traditional backpropagation algorithms [28], as they are computationally intensive, memory-demanding, and unsuitable for devices with limited resources.

An alternative for training neural networks without using backpropagation is the Forward-Forward (FF) algorithm proposed by Hinton in [29]. It replaced the backward pass with two forward passes, a positive pass using real data to maximize a “goodness” metric (sum of squared neural activities) and a negative pass using synthetic data to minimize it. While suitable for classification, generating negative data for regression remains an open challenge.

Building on this, Predictive Forward-Forward (PFP) [30] integrates predictive coding to create a stochastic, biologically plausible learning system, while μ-FF [31] replaces iterative optimization with a closed-form multivariate Ridge regression using Mean Squared Error as a loss.

Another alternative, PEPITA (Present the Error to Perturb the Input To modulate the Activity) [32], replaces the backward pass with a second forward pass where inputs are perturbed based on network error. This method maintains continuous neural activity and respects locality constraints but still requires memory to store activations and uses a fixed random matrix, complicating deployment. To address this, MEMPEPITA [33] improves memory efficiency and [34] extends the approach to deeper networks.

Kohan et al. [35] introduced a learning framework known as Signal Propagation (SP). This framework utilizes two concurrent forward passes: one for processing sensor data and another for a learning signal. However, a significant limitation of the SP framework is the lack of a clear definition for the target generator function, which produces the initial learning signal and is highly task-dependent. Despite this limitation, the paper provides several use cases and examples to illustrate the framework’s application.

Restricted Coulomb Energy (RCE) networks [36], which are hyper-spherical classifiers with dynamically initialized hidden neurons, have been explored in recent research. A recent variant, TinyRCE [37], incorporates a forgetting mechanism to prune redundant neurons, thereby enhancing memory efficiency for deployment on resource-constrained devices. Despite their categorical nature, RCE-based models are not inherently suited for regression tasks such as sensor calibration. However, their mechanism for dynamically adapting hidden neurons can inspire solutions for addressing complex tasks in an online manner.

The Resource Allocating Network (RAN) [38] leverages the concept of sequential learning to incrementally construct Gaussian RBF networks [39] in an online manner. The RAN algorithm employs a threshold to allocate new neurons. Subsequent studies, such as [40], introduced the Minimal RAN (MRAN) algorithm, which utilizes neuron activation values as the basis for pruning neurons that do not contribute significantly to the network’s performance.

RBF networks have demonstrated performance comparable to multilayer perceptron (MLP) models while offering significantly faster training speeds [39]. This efficiency makes them particularly well-suited for on-device incremental and online learning tasks, where computational resources and energy are often limited. Unlike MLPs, which rely on gradient-based optimization requiring multiple iterations, RBF networks utilize localized activation functions and straightforward learning processes, enabling faster convergence.

Single-output RBF networks were employed for pressure sensor calibration in [41], implementing periodic recalibration phases where the network structure was dynamically updated. Neurons were allocated, adjusted, or pruned based on sensor drift and performance needs. This approach resulted in a compact, online, and adaptive model capable of maintaining accuracy under varying operational conditions while preserving a low memory footprint.

RBF networks appear to be an ideal choice for edge computing scenarios that require real-time decision-making and online adaptive systems in resource-constrained environments, such as IoT, sensors, and embedded systems. Their efficient training process, ability to adapt dynamically, and low computational demands make them well-suited for these applications, where maintaining performance with limited resources is crucial.

## 3. Problem Definition

This work aims to address the question: *Is it feasible to deploy a tiny AI model that can perform both inference and training entirely deployed within the sensor package to achieve robust calibration over time, with the objective of reducing real-time errors in MEMS IMU caused by drift, aging, stress, and noise?*

The requirements for any candidate solution are the following:

- Low computational complexity that can be handled by a digital signal processor running at a maximum of 10 MHz.
- Low memory footprint using a maximum of 40 KiB (split in 8–32 KiB, respectively, for data and program) of embedded RAM memory.
- On-device learning capability, enabling both inference and online training directly inside the sensor package without requiring an external micro-processors.
- Real-time execution is achieved, with both inference and learning iterations completed within the 80 ms data acquisition interval, determined by the IMU’s operating frequency of 12.5 Hz.
- An accurate estimate of the compensation error by the sensor-embedded computing and memory assets for both accelerometer and gyroscope.
- Use the concept of catastrophic forgetting [42], where learning new information results in the loss of previously acquired knowledge, to manage complexity by pruning neurons that no longer contribute significantly to predictions.

Despite significant advances in MEMS IMU calibration techniques, existing state-of-the-art solutions have limitations that render them unsuitable for the specific problem addressed in this work, failing to meet all the requirements outlined. Classical methods, such as DLS and KF, are constrained to linear error models or necessitate external equipment, making them impractical for autonomous, embedded calibration. Although deep learning approaches achieve high accuracy, they entail substantial computational complexity and large memory footprints, often exceeding tens of thousands of parameters, which are incompatible with real-time execution and on-device learning within the strict memory and processing constraints of a digital signal processor. Furthermore, most of these methods depend on offline training using high-performance GPUs and lack the ability for online adaptation or recalibration to address sensor aging and environmental variations over time.

Therefore, there is a clear need for a lightweight, dynamic, and fully embedded solution capable of both learning and inference embodied within the sensor package. This work aims to fulfill these requirements through the proposed RBF-NN-based framework.

## 4. Contributions

This work advances the current literature on MEMS IMU calibration and on-device learning by addressing key limitations found in existing methods. While previous approaches either rely on computationally intensive deep learning models unsuitable for embedded devices or on traditional linear techniques that cannot compensate for complex, time-varying errors, this work proposes a new paradigm for real-time, online, embedded sensor self-calibration.

The primary contribution is the design and deployment of a lightweight, forward-only neural network capable of both inference and online learning entirely within the limited resources of an intelligent sensor. This work uses a dynamic neuron allocation and pruning strategy tailored for resource-constrained environments, enabling continuous recalibration without external processing. Notably, it marks a shift from traditional backpropagation-based neural networks, commonly adopted in previous studies, to a forward-only learning approach, offering a more computationally efficient and memory-friendly solution suitable for embedded, real-time applications.

Additionally, the proposed method integrates seamlessly with commercially available MEMS sensors, requiring no specialized hardware or external references, and is suitable for both static and dynamic operational conditions. By combining adaptability, efficiency, and on-device learning capabilities, this study contributes a practical and scalable solution to the ongoing challenge of autonomous MEMS IMU calibration for embedded applications.

## 5. Proposed Solution

**Figure 1:**
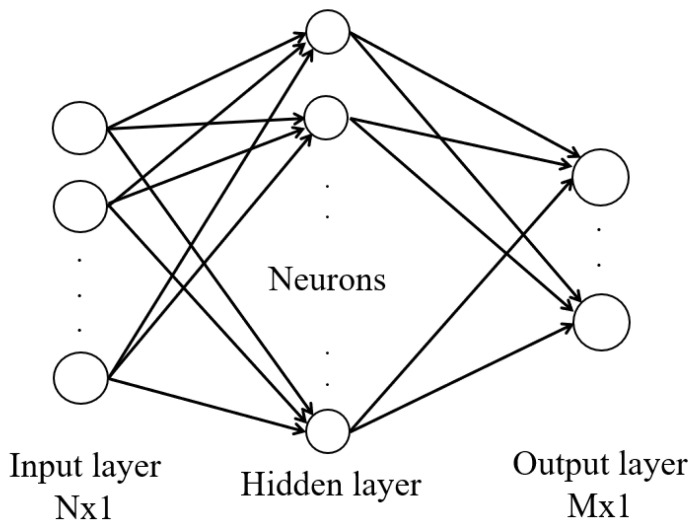
Radial Basis Function network topology: the input data consist of the uncompensated data at time *t* for each axis. The output represents the compensation error at time *t*.

To calibrate the output of the MEMS sensor, the solution is based on the RBF-NN, as proposed in [41] and shown in Figure 1. This is a forward-only fully connected network that consists of an input layer, a hidden layer, and an output layer. The input to the network is the uncompensated output from the MEMS-IMU accelerometer or gyroscope. One input neuron for each axis of the sensor is used.

**Figure 2:**
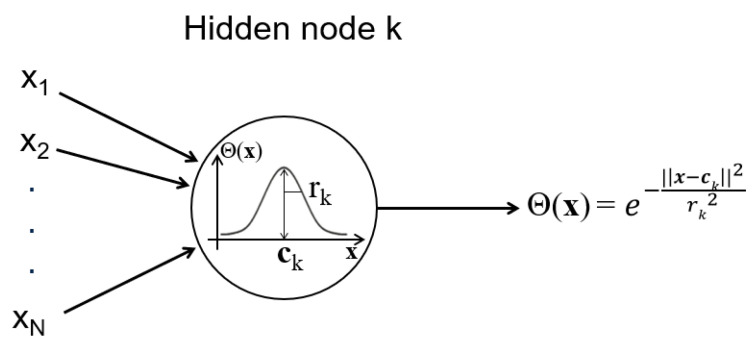
An RBF node within the hidden layer is defined by its center $c_{k}$ and radius $r_{k}$, which correspond to the mean and standard deviation of the Gaussian function, respectively. Each hidden node calculates its activation based on the input values (the uncalibrated output of the sensor) that compose the input vector **x**. The computed activation values reflect the node’s response to the specific input and are subsequently transmitted to each output neuron.

Each input neuron is connected to every neuron in the hidden layer. Figure 2 provides a detailed view of a neuron from the hidden layer. Each neuron has a its center ($c_{k}$) and radius ($r_{k}$) and uses the Gaussian activation function. The center and the standard deviation of the Gaussian function are, respectively, $c_{k}$ and $r_{k}$. The activation function $\theta_{k}\left(x\right)$ of each neuron k is computed as follows:

(1) $$\theta_{k}\left(x\right)=e^{-\frac{∥x-c_{k}{∥}^{2}}{r_{k}^{2}}}$$

The number of neurons in the hidden layer is not predefined because the neurons are dynamically allocated and pruned during the learning stage. Three output neurons (Figure 1) are used by the inference model to compute the compensation errors, which are then utilized to compensate the MEMS output.

### 5.1. Learning Phase

The learning phase of an RBF-NN is divided into an offline phase and an online phase.

In the offline phase, the network’s centers, radii, and weights are initialized using the k-means algorithm [43]: N cluster centroids are computed and used as RBF initial node centers $c_{k}$. The radius $r_{k}$ of the k-th hidden node is initialized as the distance to the nearest center, as follows:

(2) $$r_{k}=∥c_{k}-c_{m^{\prime }}∥,\;m^{\prime }=\arg\min_{1\leq m\leq N,m\neq k}∥c_{k}-c_{m}∥$$

Equation (2) identifies the center $m^{\prime }$ among all N available, excluding the k-th, which has the minimum distance to the k-th center. This distance is then used to initialize the radius of the k-th neuron.

The weight matrix W of the connections between the hidden layer and the output layer is initialized by solving a linear optimization problem using the least squares method [39]. In this context, Y is a matrix that contains the output data in the training set in each row, and Ω is a matrix whose rows are the activations of the input data computed using the Gaussian function as the nonlinear activation function, as described in Equation (1). The weight matrix W is then computed as follows:

(3) $$W={\left(\Omega^{T}\Omega \right)}^{-1}\Omega^{T}Y$$

In addition to the N nodes, a bias node is also considered, with activation equal to 1.

The online phase is performed periodically and follows this idea: for every input value **x**(t), if the prediction error **e**(t) (as defined in Equation (5)) and the distance of **x**(t) from the nearest neuron (defined in Equation (6)) exceeds two predefined thresholds, a new neuron is allocated; otherwise, the existing neurons are updated. At the end of each learning iteration, it is determined whether any neurons need to be pruned.

Thus, this phase consists of three fundamental steps: allocation, update, and pruning.

#### 5.1.1. Allocation of New Neurons

Firstly, the network computes the output ${\hat{y}}_{i}\left(x\left(t\right)\right)$ for every i-th input, using the activation $\theta_{k}\left(x\left(t\right)\right)$ computed as in Equation (1):

(4) $${\hat{y}}_{i}\left(t\right)=\omega_{0,i}+\sum_{k=1}^{N}\omega_{k,i}\theta_{k}\left(x\right)$$

Subsequently, computes the prediction error:

(5) $$e\left(t\right)=y\left(t\right)-\hat{y}\left(t\right)$$

And the distance of the new input value from the nearest neuron:

(6) $$d\left(t\right)=\min_{1\leq k\leq N}∥x\left(t\right)-c_{k}∥$$

Finally, if the error **e**(t) exceed the error threshold ϵ and the distance d(t) exceeds the distance threshold δ, a new neuron has to be allocated with the following characteristics, where *k* represents the overlap factor:


{(7)CN+1=x(t)(8)rN+1=k·d(t)(9)ωN+1=e(t)

The center of the new neuron is placed in the position of the input value. The radius is determined by multiplying the distance of the new input from the nearest neighbor by the overlap factor. The new values in the weight matrix are set equal to the prediction error.

#### 5.1.2. Update Existing Neurons

When the allocation condition is not verified, the network does not add any neurons to its topology but updates the centers and the weights of the existing ones. Specifically, $\omega_{0}$ represents the weights of the bias term, η is the learning rate, $\omega_{k,i}$ denotes the weight between the hidden layer *k* and the output layer *i*, and $c_{k}$ represents the centers of the neurons. The updates are computed as follows:


{(10a)ω0←ω0+ηe(t)(10b)ωk,i←ωk,i+ηei(t)θk(t)(10c)ck←ck+η2θk(x(t))rk2(e(t)·ωk)(x(t)−ck)

The weights in Equations (10a) and (10b) are updated by adding the product of the error gradient with respect to them and the learning rate, while the centers in Equation (10c) are updated by adding the product of the learning rate and the error gradient with respect to the center.

#### 5.1.3. Pruning of Useless Neurons

At the end of every learning iteration, the normalized activation of every hidden neuron $\theta_{k}^{\mathrm{norm}}x\left(t\right)$ is computed as follows:

(11) $$\theta_{k}^{\mathrm{norm}}\left(x(t)\right)=\left|\frac{\theta_{k}\left(x(t)\right)}{\max_{k}\theta_{k}\left(x(t)\right)}\right|$$

Subsequently, if a neuron has not been sufficiently activated—specifically if the quantity $\theta_{k}^{\mathrm{norm}}x\left(t\right)$ remains below a predefined threshold α for more than *W* consecutive iterations, where *W* denotes the pruning window—then the neuron is subject to removal.

This pruning strategy is crucial for preventing uncontrolled growth in the number of neurons within the hidden layer and ensuring that the network remains efficient by eliminating neurons that do not significantly contribute to its predictive performance. It leverages the concept of catastrophic forgetting [42], typically viewed negatively in neural networks, to discard knowledge that has become obsolete due to environmental changes, drift, or other factors. This approach is particularly important for sensors with limited memory capacity, as it helps maintain optimal performance by freeing up valuable space.

### 5.2. Inference Phase

When the neural network is not engaged in the periodic training process, it is ready for the inference phase. In this stage, given an input vector **x**(t), each element of this vector is assigned to the corresponding neuron in the input layer. Subsequently, the activation of each neuron in the hidden layer is calculated using Equation (1). Finally, the output values are determined by each output neuron using Equation (4).

It is important to note that during the inference phase, the topology of the network remains static. Neurons are neither updated, allocated, nor pruned.

## 6. Deployment on Device

The Intelligent Sensor Processing Unit (ISPU) [44] is an ultra-low-power, high-performance programmable core with high computational efficiency. It is equipped with 32 KiB of program RAM and 8 KiB of data RAM.

**Figure 3:**
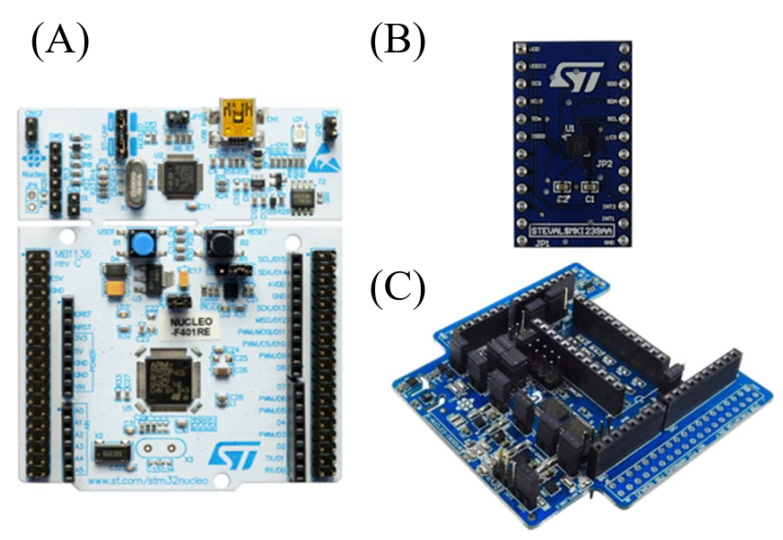
Hardware configuration for the deployment of the model inside the ISPU. Figure (**A**) is the NUCLEO F410RE Development Board, and Figure (**B**) is the STEVAL-MKI299A adapter board, which contains the LSM6DSO16IS IMU with the ISPU integrated. Figure (**C**) is the X-NUCLEO-IKS01A3 motion MEMS and environmental sensor expansion board.

The ISPU is integrated into the LSM6DSO16IS sensor (Figure 3B), a six-axis Inertial Measurement Unit (IMU). It includes six internal sensors integrated into a single package: a three-axis digital accelerometer and a three-axis digital gyroscope, as well as the ability to act as the master of four external sensors: a magnetometer, pressure sensor, humidity sensor, and temperature sensor placed on the expansion module (Figure 3C).

The ISPU core is built upon the STRED [45], a proprietary reduced energy instruction set micro-architecture developed by ST. The ISPU toolchain facilitates the development of C code and enables the loading of custom programs into the core as long as they fit within the available memory constraints.

The LSM6DSO16IS is connected to the system via the STEVAL-MKI299A adapter board (Figure 3B), which eases the hardware integration. The X-NUCLEO-IKS01A3 (Figure 3C) serves as the motion MEMS and environmental sensor expansion board, providing additional sensor interfaces. The overall system operation is managed by the NUCLEO F410RE (Figure 3A) development board, based on the STM32F410 MCU. This MCU enables the communication between the PC and the RBF-NN embedded into the ISPU and does not perform any AI operation as far as this deployment is concerned.

The RBF-NN code has been flashed into the MCU FLASH using the tool STM32Cube IDE. At boot time, the code is then copied into the program RAM (32 KiB maximum) of the ISPU, from where the instructions are executed. This is because ISPU doesn’t embody any FLASH memory.

Table 1 presents the memory occupation of two versions of the RBF-NN (the 32-bit floating point and the quantized 16-bit integer) within the ISPU memory. Both implementations occupy less than 21 KiB of the 40 KiB available in the ISPU, with a maximum of 1 KiB for data RAM and less than 20 KiB for program RAM. The fp32 version occupies less program RAM since it features more compact C code, while the int16 version includes additional code for quantization–aware training [46]. As expected, the fp32 version requires nearly double the data RAM since its data representation uses twice the bits of the int16 version.

## 7. Datasets

In this section, the two datasets utilized in this work are described. The first comprises only accelerometer and gyroscope raw data with ground truth measures. The second used more devices to perform attitude estimation of the drone on which the IMU was mounted.

### 7.1. Data Collection in the Field

**Figure 4:**
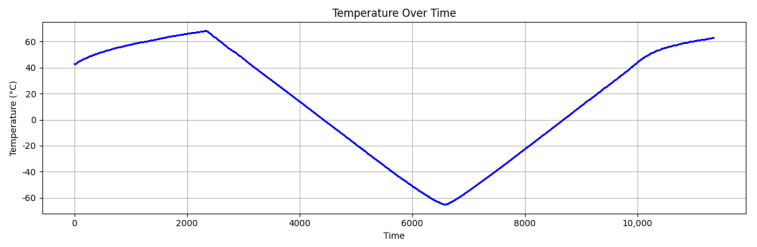
Temperature measured by the first DUT during the data collection.

The first dataset was composed of real IMU data collected in a collection campaign carried out in [23]. The LSM6DSV six-axis IMU-MEMS, comprising a three-axis digital gyroscope, a three-axis digital accelerometer, and a digital temperature sensor, was used. 21 IMUs were posed horizontally on the dedicated PCB and collected data for 3 h and 15 min each under thermal stress provided using a Temptronic ThermoStream system [47] with temperatures ranging from −40 °C to 85 °C, as shown in Figure 4, with a gradient of 2 °C/min. Each device collected data with an output data rate of 15 Hz, meaning that it read values approximately every 66 ms.

**Figure 5:**
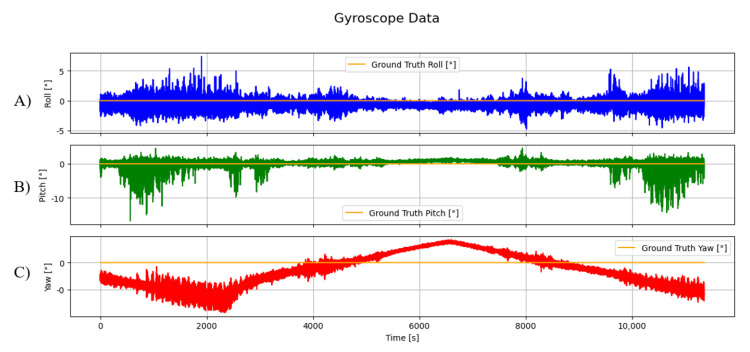
Data of roll (**A**), pitch (**B**), and yaw (**C**) read by the gyroscope of the first DUT, represented in °, with corresponding ground truth values shown in orange.

In Figure 5, the values are shown read by the first device under test (DUT) of the gyroscope. All The DUTs were placed in a steady horizontal position, and the values measured should be 0° for all roll, pitch, and heading.

**Figure 6:**
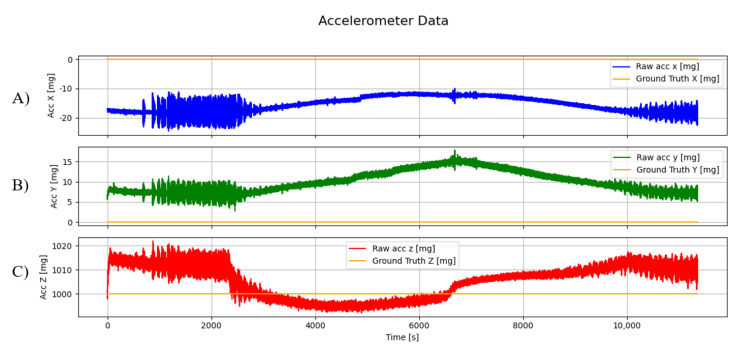
Accelerometer data of the first DUT represented in mg along the three axes: (**A**) x-axis (blue), (**B**) y-axis (green), and (**C**) z-axis (red), with corresponding ground truth values shown in orange.

In Figure 6 are shown the values read by the accelerometer of the first DUT. The devices were in a steady horizontal position, meaning that the values read should be 0 mg on the x-axis and y-axis and 1000 mg on the z-axis.

### 7.2. EuRoC MAV Dataset

The EuRoC Micro Aerial Vehicle (MAV) Dataset, curated by the Autonomous Systems Lab at ETH Zurich [48], represents a valuable benchmark for advancing research in robotics, particularly in the areas of autonomous navigation, visual–inertial odometry, and mapping. This dataset was carefully designed to support the development and rigorous validation of algorithms for micro aerial vehicles, offering a comprehensive set of multi-modal sensor data collected in realistic, diverse operational scenarios.

The dataset comprises eleven sequences recorded across three distinct environments: two industrial machine halls and a furnished apartment setting. Each sequence integrates data from multiple onboard sensors, including stereo cameras, an Inertial Measurement Unit (IMU), and, in selected sequences, a laser range finder. The stereo cameras capture high-resolution images essential for perception and mapping tasks, while the IMU supplies inertial measurements critical for estimating motion and maintaining vehicle stability during flight.

The IMU data, which are of primary interest in this study, include linear acceleration and angular velocity measurements acquired from an ADIS16448 MEMS IMU at a frequency of 200 Hz. These high-frequency inertial measurements enable the detailed characterization of fast dynamic motions, making the dataset particularly suitable for the development of accurate visual–inertial navigation systems. Notably, unlike the other dataset employed in this work (where IMU data were collected with the sensor in a static, horizontal position), the EuRoC MAV Dataset captures measurements under varying and dynamic conditions. The MAV operates in motion throughout all eleven sequences, performing translational and rotational movements and assuming different spatial orientations as it navigates through the environments. This results in gyroscope data that reflect a broad range of angular velocities, corresponding to both stationary and in-motion states, thereby providing a richer and more challenging context for attitude estimation.

In addition to the raw sensor data, the dataset includes precise ground truth poses provided by a motion capture system in the machine hall environments and a Leica MS50 laser tracker in the apartment setting. These ground truth annotations, expressed as position and orientation values in the form of quaternions, are essential for supervised learning approaches and serve as the reference against which model predictions for attitude estimation are evaluated by this work.

## 8. Metrics

Two metrics have been adopted to measure the results of the proposed fp32 and int16 models with the two different datasets.

### 8.1. Field Data

To measure the error reduced by the RBF-NN and by the benchmark model [23], the error reduction percentage is used. The operands are defined as follows:

- $MAE_{MEMS}$: the Mean Absolute Error of the uncalibrated MEMS IMU.
- $MAE_{Model}$: the MAE of the sensor after being calibrated by the models under study.
- The MAE is computed when the ground truth reference is available.

The error reduction percentage is then computed as

(12) $$\mathrm{Error}\;\mathrm{Reduction}\;\left[\%\right]=\frac{MAE_{MEMS}-MAE_{Model}}{MAE_{MEMS}}\times 100$$

### 8.2. EuRoC MAV

To quantitatively assess the performances of the RBF-NN against traditional methods, the Absolute Orientation Error (AOE) [22] has been used. The operands are defined as follows:

- $R_{n}\in SO\left(3\right)$: the rotation matrix at timestamp *n* that maps the body coordinate frame to the navigation coordinate frame.
- ${\hat{R}}_{n}\in SO\left(3\right)$: the estimated value of the rotation matrix.
- *L*: the sequence length.
- log(·): the SO(3) logarithm map.

The AOE computes the mean square error between the ground truth and the estimated orientation, and it is described as follows:

(13) $$\mathrm{AOE}=\sqrt{\frac{1}{L}\sum_{n=1}^{L}{∥\log\left(R_{n}^{T}{\hat{R}}_{n}\right)∥}_{2}^{2}}$$

## 9. Experiments and Results

### 9.1. Hyper-Parameters Tuning

The RBF-NN model includes several hyper-parameters that are of fundamental importance for its performance and must be carefully optimized according to the specific problem and the scale of the input data. Among these, three critical thresholds play a central role:

- Error threshold; (ϵ)
- Distance threshold (δ);
- The activation threshold (α), used in the pruning strategy to assess whether a neuron’s activation is sufficiently significant to be retained for prediction during a given iteration.

Additional key hyper-parameters include the learning rate (η), which is essential for updating the weights between the hidden and output layers; the pruning window (*W*), which defines the number of consecutive iterations in which a neuron remains inactive before being pruned; and the overlap factor (*k*), which influences the radius assigned to a newly allocated neuron.

The hyperparameter optimization process was conducted using Optuna [49], leveraging a Bayesian optimization search algorithm [50] to efficiently explore the hyperparameter space. As a result, three sets of optimal hyperparameter values were identified: two for the Field Dataset—one for accelerometer data and one for gyroscope data—and one for the EuRoC MAV Dataset:

- Field Data Accelerometer Calibration: *k* = 1.98, ϵ = 5.32, η = 0.094, α = 0.189, *W* = 492, δ = 9.49.
- Field Data Gyroscope Calibration: *k* = 1.46, ϵ = 1.42, η = 0.097, α = 0.012, *W* = 88, δ = 3.37.
- EuRoC MAV Data: *k* = 1.28, ϵ = 1.15, η = 0.033, α = 0.88, *W* = 324, δ = 2.0.

### 9.2. Field Data

**Figure 7:**
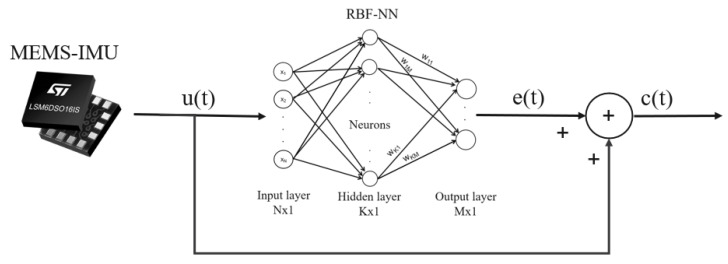
The uncompensated output of the MEMS-IMU u(t) is given as input to the RBF-NN, which predicts the error of the sensor. This error e(t) is then added to the uncompensated measure u(t) to produce the compensated output of the calibrated sensor c(t).

To evaluate the performance of the RBF-NN on the dataset described in Section 7.1, the system architecture employed corresponds to the configuration illustrated in Figure 7. The RBF-NN processes the raw data from the MEMS-IMU accelerometer as input, utilizing three neurons in the input layer, each corresponding to one of the three axes. The network estimates the associated sensor error through one output neuron for each axis and adds this estimated error to the raw measurement to produce the compensated sensor output. The number of neurons in the hidden layer, on the other hand, is not predetermined but changes dynamically during the learning phases.

The proposed model has been compared against a compact CNN previously utilized for this purpose in [23]. Specifically, the benchmark model adopted for comparison is the Paral Conv 1D architecture, identified in [23] as achieving the highest performance on this dataset. This comparison was designed to emphasize the relative advantages and limitations of a conventional lightweight backpropagation-based model versus a forward-only architecture, as exemplified by the RBF-NN. The Paral Conv 1D model is shown in Table 2.

Both the Paral Conv 1D and the RBF-NN models were trained and tested using data collected from 21 DUTs. Unlike the approach presented in [23], the models in this study were trained to incorporate periodic recalibrations at varying recalibration frequencies.

The training of the Paral Conv 1D model was conducted over 200 epochs with a batch size of 32. The selected hyperparameters for this model include a learning rate of 0.01, the Adam optimizer, and an input training window overlap of 0.5. Similar to the RBF-NN (as shown in Figure 7), the Paral Conv 1D model is designed to predict the sensor errors, which are subsequently applied to correct the raw sensor measurements.

**Figure 8:**
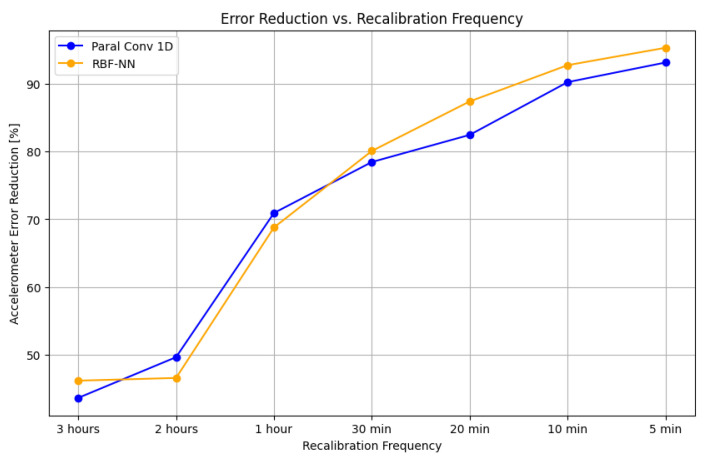
This graph shows the error reduction percentage achieved from the accelerometer Field Data at different recalibration frequencies for the two models under comparison: Paral Conv 1D and RBF-NN.

Figure 8 reports the accelerometer error reduction percentages achieved by the Paral Conv 1D and RBF-NN models across various recalibration frequencies. As expected, both models exhibit a progressive decline in error reduction performance as the recalibration interval increases. However, RBF-NN model consistently outperforms the Paral Conv 1D model, particularly at higher recalibration frequencies. Specifically, when recalibration occurs every 5 min, the RBF-NN achieves an error reduction of 95.27%, higher than the 93.10% reduction achieved by the Paral Conv 1D model. This performance advantage persists at 10 min and 20 min intervals, with the RBF-NN maintaining higher error reduction rates of 92.71% and 87.37%, respectively, compared with 90.21% and 82.44% for Paral Conv 1D. As the recalibration frequency decreases beyond 30 min, both models experience a notable drop in performance; however, the performance gap between them diminishes, converging to similar error reduction values at 3-hour intervals (46.23% for RBF-NN and 43.69% for Paral Conv 1D).

**Figure 9:**
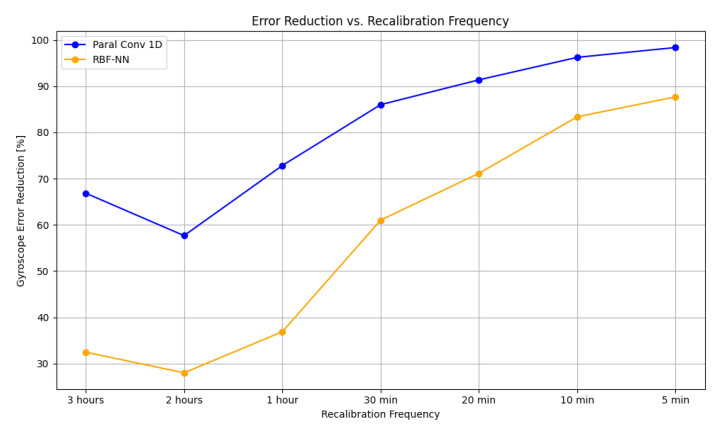
This graph shows the error reduction percentage achieved from the gyroscope Field Data at different recalibration frequencies for the two models under comparison: Paral Conv 1D and RBF-NN.

In Figure 9, the error reduction in the RBF-NN compared with the Paral Conv 1D model on the gyroscope data from the Field Dataset is illustrated. The Paral Conv 1D model achieves an error reduction ranging from 57% to 98%, whereas the RBF-NN performs less effectively than the traditional approach, with error reduction results between 32% and 88%. The disparity between the models diminishes as the recalibration frequency increases. When the recalibration is performed every 1 h, the gap of error reduction between the models is around 30%, while when it is performed every 5 min, it decreases to 10%.

#### Performances on the ISPU

The Paral Conv 1D model proposed in [23] was implemented on the ISPU platform, with the corresponding experimental results presented in Table 3. It is worth emphasizing that the RAM and ROM memory figures reported for the Paral Conv 1D account solely for the parameter activation and weights memory, as specified in [23]. In contrast, the RAM and ROM requirements for the RBF-NN also include the memory footprint of the code required to implement the network’s structure, as well as the learning and inference workloads.

The number of parameters and the multiply-accumulate operations (MACC) required by the RBF-NN are expressed as a range, reflecting the variability in the number of neurons dynamically instantiated in the hidden layer. This range represents the minimum and maximum values observed across the experiments reported in Figure 8 and Figure 9.

Notably, the Paral Conv 1D model features more than 17 times the number of trainable parameters used by the RBF-NNs tested in this study and demands over 5 times the number of MACC operations. Furthermore, the Paral Conv 1D was unable to meet real-time execution constraints, as its inference time exceeded the 80 ms interval required by a sensor with 12.5 Hz of output data rate to acquire a new data triplet. Conversely, the RBF-NN demonstrated the capability to perform inference within real-time constraints, as reported in Table 3, and also to complete each learning iteration within the same 80 ms window. While the learning times are not explicitly listed in the table, experimental observations confirmed that they consistently remained below this threshold.

### 9.3. EuRoC MAV

The RBF-NN has been used to calibrate the gyroscope measurements of the EuRoC MAV Dataset and perform attitude estimation. The model has been trained and tested on the EuRoC MAV Dataset, and the results have been compared with the models described in [18,19,22], using the AOE as a metric, as described in Section 8.2.

**Figure 10:**
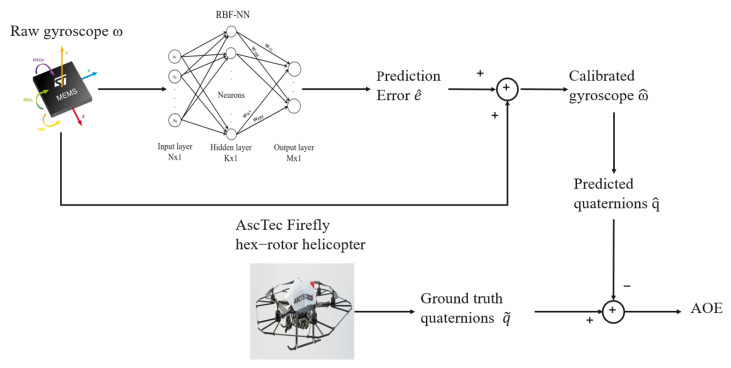
System Architecture of the model used for evaluation of the RBF-NN on the EuRoC MAV Dataset.

The RBF-NN, as shown in Figure 10, took as input the raw gyroscope values of the IMUs and predicted the error of the sensor, which was subsequently added to the raw value to compute the calibrated measure. This RBF-NN, similar to the one used for accelerometer calibration in Section 9.2, consists of three neurons in the input layer and three in the output layer, one for each axis. The number of neurons in the hidden layer, however, adjusts dynamically during the training process.

From the calibrated measures of the gyroscope, the quaternions are computed, as conducted in [22]. Quaternions are used to describe the orientation of the aerial vehicle at each moment in time. From the predicted quaternions and the ones of the ground truth are computed the rotation matrices, used to compute the AOE. In this way, the RBF-NN is performing gyroscope calibration and also attitude estimation of the MAV.

Differently from what has been done with the Field Dataset, in this case, the RBF-NN has not been trained and tested with periodic recalibrations but, for consistency reasons with the benchmark models, has been trained on six of the eleven sequences of the EuRoC MAV dataset and tested with the remaining five, as shown in Table 4. The training dataset had a total of 146,966 IMU measurements, while the testing dataset had 122,475.

Table 5 presents the comparative evaluation of AOE, expressed in degrees, across the test sequences derived from the EuRoC MAV dataset. The performance of the proposed RBF-NN model is assessed against several established methods: DIG [19], OriNet [18], and two configurations of TinyGC-Net [22] (Calibrated and Denoised). The best results in each row are highlighted in bold.

The results indicate that the RBF-NN achieves competitive orientation estimation accuracy, with an average AOE of 2.84°, positioning it as the second-best performing model after DIG (2.09°). Notably, the RBF-NN consistently outperforms the other methods, including both configurations of TinyGC-Net and OriNet, across the majority of the individual test sequences. For instance, in the V1_01_easy sequence, the RBF-NN achieves an AOE of 2.79°, closely approaching the performance of DIG while remaining significantly superior to OriNet (8.36°) and both TinyGC-Net variants (5.32° and 4.16°). This trend is similarly observed across other sequences, reflecting the model’s robustness in varying environmental conditions and motion profiles. The RBF-NN is the best on the V2_02_medium sequence, which can be considered as one of the most difficult, achieving an AOE of 3.13°, while the second best is DIG with 3.85°.

Moreover, the RBF-NN, like TinyGC-Net, uses only the data acquired by the gyroscope and not the data of the accelerometer.

In terms of resource efficiency, the RBF-NN demonstrates a balanced trade-off between model complexity and accuracy. With 37 parameters, it requires slightly more parameters than TinyGC-Net (Calibrated) (27 parameters), yet considerably fewer than TinyGC-Net (Denoised) (195 parameters) and OriNet (77,052 parameters). This parameter efficiency, combined with its deployment capability on the ISPU, with both training and inference operating on it rather than a GPU, underscores the model’s suitability for real-time, on-device applications in resource-constrained embedded environments.

## 10. Discussion

This proposed approach to real-time MEMS IMU sensor self-calibration has demonstrated its effectiveness under a variety of operational conditions, including both static controlled field data and dynamic real-world dataset as well as environments subject to thermal stress, and has been successfully applied to both accelerometer and gyroscope data. The results achieved validate the suitability of the RBF-NN for performing real-time, adaptive calibration within the highly constrained environment of an embedded digital signal processor.

This work is a proven demonstration that forward-only learning networks, such as the proposed RBF-NN, can achieve competitive calibration performance compared with lightweight CNNs while significantly reducing the memory footprint and computational demand.

Furthermore, beyond its primary application in sensor calibration, the method has achieved competitive results in attitude estimation tasks for micro aerial vehicles on the EuRoC MAV dataset, confirming its versatility and robustness. The model has been compared with more complex, GPU-trained models and, despite its compact size, has provided competitive results.

The deployment of the ISPU further demonstrated the feasibility of executing both learning and inference within stringent program and data memory budgets. The quantized 16-bit integer implementation, in particular, ensured compatibility with embedded hardware constraints while preserving performance, confirming the suitability of quantization techniques for TinyML deployments.

Despite the strong results, some limitations of this work should be acknowledged. The model currently focuses on calibrating accelerometer and gyroscope measurements independently without explicitly considering multi-sensor fusion strategies that could leverage redundant information from other available sensor modalities. Additionally, while the dynamic allocation and pruning mechanisms successfully control memory growth, their long-term stability and behavior under highly non-stationary conditions warrant further investigation.

## 11. Conclusions

This study introduced an innovative, lightweight, on-device learning framework for the real-time self-calibration of MEMS-based IMUs employing Radial Basis Function Neural Networks. The proposed approach demonstrated the feasibility of deploying a dynamically adaptable neural network topology entirely within the ISPU, effectively addressing the memory and computational constraints inherent to such embedded platforms. Experimental results obtained from field-acquired data under thermal stress conditions indicated that the RBF-NN was capable of reducing accelerometer error by between 46% and 95%, consistently outperforming a benchmark tiny CNN model. Although the gyroscope calibration results showed the RBF-NN performing marginally below the CNN at lower recalibration frequencies, it progressively closed this performance gap as recalibration intervals were shortened.

Furthermore, evaluations on dynamic datasets, including the EuRoC MAV, revealed that the RBF-NN achieved an average AOE of 2.84°, positioning it competitively against more complex, GPU-trained models such as OriNET, DIG, and TinyGC-Net while requiring orders of magnitude fewer parameters and computational resources. The RBF-NN successfully maintained real-time performance within the stringent memory and timing constraints of the ISPU, utilizing only 37 parameters and completing both inference and online learning processes within 80 ms. Overall, this work substantiates the feasibility, efficiency, and adaptability of edge AI methodologies for real-time MEMS sensor self-calibration and attitude estimation, providing a reliable, low-power, real-time alternative to conventional calibration techniques and resource-intensive deep learning models in embedded applications.

## Figures and Tables

**Table 1:** KBytes occupied by the two different RBF-NN versions (fp32 and int16) inside the ISPU.

	Program RAM [KiB]	Data RAM [KiB]
ISPU Availability	32	8
fp32 RBF-NN	18.53	1.02
int16 RBF-NN	19.74	0.637

**Table 2:** Parallel Conv1D model summary. The model is composed of 3 parallel branches, which outputs concatenate together in the ‘Concatenate’ layer.

Layer (Type)	Output Shape	Kernel Size	Connected to	Branch	Parameters
Input (InputLayer)	(50, 3)	–	[]	–	0
Conv1D 1 (Conv1D)	(50, 8)	3 × 1	[Input]	1	80
Conv1D 2 (Conv1D)	(50, 8)	4 × 1	[Input]	2	104
Conv1D 3 (Conv1D)	(50, 8)	5 × 1	[Input]	3	128
Conv1D 4 (Conv1D)	(50, 4)	3 × 1	[Conv1D 1]	1	80
Conv1D 5 (Conv1D)	(50, 4)	3 × 1	[Conv1D 2]	2	100
Conv1D 6 (Conv1D)	(50, 4)	3 × 1	[Conv1D 3]	3	100
Concatenate	(50, 12)	–	[Conv1D 4, Conv1D 5, Conv1D 6]	–	–
Conv1D 7 (Conv1D)	(50, 3)	1 × 1	[Concatenate]	common	39
Total parameters:					651

**Table 3:** Resource consumption and inference time of the Paral Conv 1D model in the ISPU set at 10 MHz operative frequency.

	Parameters	RAM [KiB]	ROM [KiB]	MACC (per Sample)	Inference Time ISPU @ 10 MHz [ms]
Paral Conv 1D	651	4.68	2.54	649	173.7
RBF-NN	[10, 38]	18.53	1.02	[35, 115]	7.27

**Table 4:** Train and Test Splits of EuRoC MAV Dataset.

Train Dataset	Test Dataset
Machine_Hall01-Easy	Machine_Hall02-Easy
Machine_Hall03-Medium	Machine_Hall04-Difficult
Machine_Hall05-Difficult	Vicon_Room_1_03-Difficult
Vicon_Room_1_02-Medium	Vicon_Room_2_02-Medium
Vicon_Room_2_01-Easy	Vicon_Room_1_01-Easy
Vicon_Room_2_03-Difficult	

**Table 5:** AOE in degree on the test sequences.

	Raw	DIG [19]	OriNet [18]	TinyGC-Net [22] (Calibrated)	TinyGC-Net [22] (Denoised)	RBF-NN
MH_02_easy	146	**1.39**	5.75	6.36	4.60	3.23
MH_04_difficult	130	**1.40**	8.86	5.32	4.16	2.20
V1_01_easy	71.3	**1.13**	8.36	7.12	6.27	2.79
V1_03_difficult	119	2.70	14.70	3.64	**2.23**	2.85
V2_02_medium	117	3.85	11.70	4.95	4.10	**3.13**
Average	116.66	**2.09**	9.87	5.48	4.27	2.84
parameter count	-	77,052	-	**27**	195	37
model input	-	ACC & GYRO	ACC & GYRO	GYRO	GYRO	GYRO
training platform	-	GPU	GPU	GPU	GPU	ISPU
deployment platform	-	GPU	GPU	MCU	MCU	ISPU

## Data Availability

Field Dataset is an internal dataset of STMicroelectronics. EuRoC MAV dataset is a public available dataset at https://projects.asl.ethz.ch/datasets/doku.php?id=kmavvisualinertialdatasets, Date of Access 11/6/2025.

## Abbreviations

ACC	Accelerometer
ADAS	Advanced Driver-Assistance Systems
AOE	Absolute Orientation Error
ANN	Artificial Neural Network
CNN	Convolutional Neural Network
DLS	Direct Least Squares
DL	Deep Learning
DSP	Digital Signal Processor
DUT	Device Under Test
EuRoC MAV	European Robotics Challenges Micro Aerial Vehicle
FF	Forward-Forward
GYRO	Gyroscope
GPU	Graphics Processing Unit
GRU	Gated Recurrent Unit
IMU	Inertial Measurement Unit
ISPU	Intelligent Sensor Processing Unit
KF	Kalman Filter
KiB	Kibibyte (1024 bytes)
LSTM	Long Short-Term Memory
MACC	Multiply-Accumulate Operations
MAV	Micro Aerial Vehicle
MAE	Mean Absolute Error
MCU	Microcontroller unit
MEMPEPITA	Memory-efficient PEPITA
MEMS	Micro-Electro-Mechanical Systems
MRAN	Minimal Resource Allocating Network
NAS	Neural Architecture Search
PCB	Printed circuit board
PEPITA	Error-Driven Input Modulation Learning Algorithm
PFP	Predictive Forward-Forward
RAM	Random Access Memory
RAN	Resource Allocating Network
RBF	Radial Basis Function
RBF-NN	Radial Basis Function Neural Network
RCE	Restricted Coulomb Energy (network)
ROM	Read-Only Memory
RTE	Real-Time Execution
SO(3)	Special Orthogonal Group in 3D (rotation matrices)
SRU	State Reset Unit Neural Network
STRED	STMicroelectronics RISC Embedded Design
TCN	Temporal Convolutional Network
TUKF	Transformed Unscented Kalman Filter
